# The Open Body Closed: A Rationale for the Abandonment of Bloodletting, Based on Nineteenth-Century Swedish Medicine

**DOI:** 10.1093/shm/hkae069

**Published:** 2024-11-21

**Authors:** Annelie Drakman

**Affiliations:** History of Ideas, Department of Culture and Aesthetics, Stockholm University, Stockholm, Sweden

**Keywords:** bloodletting, Sweden, flow-managing medicine, open/closed body, humoralism

## Abstract

This article contains an analysis of the use and abandonment of bloodletting in Sweden 1820–1900. Close readings of over 8,000 yearly reports by Swedish provincial doctors and popular medical handbooks, journals and notes from medical societies have been used, as well as key word searches meant to illustrate overarching tendencies. One result is that quantitative balance between humours was not an aim of therapeutic bleeding in this context. Rather, bloodletting was mainly used to reinstate regular flows in a hydraulic model of the body. It is argued that a shift from focusing on smooth flows to seeing bleeding as blood loss marked a transformation of the medical imagination from working with an ‘open’, malleable body to a ‘closed’, fixed body. This helps explain why therapeutic bleeding, for millennia the most important practice in medical practitioners’ arsenal, was silently abandoned decades before the breakthrough of bacteriology and scientific medicine.

Bloodletting was a signature practice for medicine in the early nineteenth century. This period was characterised by energetic support for phlebotomy and leeching in Europe and the United States by followers of ‘heroic medicine’, championed by doctors such as Benjamin Rush (1746–1813) in Philadelphia and Francois-Joseph-Victor Broussais (1772–1838) in Paris.^1^ Tens of millions of leeches were moved between nations yearly as a part of the lively leech trade, for instance, leading to the use of leeches in Dublin increasing 45-fold between 1780 and 1836.^2^ The journal *The Lancet*, today one of the most influential in the world, took its name from the instrument considered most characteristic of medicine in its inaugural year 1823: the bloodletting knife.^3^

Towards the end of the century, however, very few doctors were still bleeding their patients. The process was gradual, but as the historian of medicine John Harley Warner points out in *The Therapeutic Perspective*—the most thorough investigation of nineteenth-century phlebotomy to date—the reduction in the use of therapeutic bleeding between the 1820s and the 1880s was ‘dramatic’, even ‘momentous’.^4^ Historian Charles Rosenberg calls this abandonment a ‘therapeutic revolution’.^5^ Why was a therapy which had been considered effective for millennia, and which was more popular than ever before, abandoned without hardly any debate decades before the breakthrough of bacteriology and scientific medicine? For bloodletting to be abandoned, a far-reaching change in the medical imagination must have taken place. As historian of the body Shigehisa Kuriyama puts it: ‘The history of bloodletting poses two basic problems. One is the puzzle of bloodletting’s traditional popularity; the other is the enigma of its modern decline’.^6^ What exactly was it that was being re-thought?

Historians have pondered the use and abandonment of bloodletting in nineteenth-century Western medicine for decades. Several classical texts from the 1970s and 1980s still remain relevant, and the question has also attracted interest from scholars outside of the history of medicine field in more recent years, such as David Wootton and K. Codell Carter.^7^

Several reasons for the abandonment of bloodletting have been offered, generally on the themes of increased rationality and social pressure. But as Warner points out, the question is complex:

Historians have frequently listed the factors that contributed to the demise of therapeutic bloodletting. These include French sceptical empiricism, growing faith in the healing power of nature, pressure from patients, and the introduction of alternative treatments. Competition from such sectarian practitioners as homeopathists... and Thomsonians... and their derision of heroically, aggressively used orthodox therapies such as bloodletting... have also commonly been cited. All of these factors did indeed contribute to the decline in bloodletting. Yet at the same time each one also encouraged regular physicians to defend the practice even more stridently than before and to cling to it as a symbol of the orthodox profession. To understand why therapeutic change occurred and what it meant, therefore, the historian must scrutinize and explain the process of change, not merely describe its animi and end points.^8^

This article is meant to supplement the explanations presented by Warner and others with a new rationale for both the use and abandonment of therapeutic bleeding. The main argument is that early nineteenth-century bloodletting, at least in the Swedish case, was motivated not by humoralism but by a hydraulic view of the body as characterised by steady, regular flows of bodily fluids. To a large extent, therapeutic intervention sought to modify circulation into, within and out from the body. After the mid-1800s, however, doctors instead started understanding the body as closed off and autonomous from its environment, and began trying to protect its boundaries. Flows into the body began to be seen as attacks, flows within the body became mostly irrelevant and flows out from the body were treated as dangerous losses of energy. This transition from ‘flow-managing’ to ‘boundary-protecting’ medicine was, I argue, a reason why bloodletting lost favour among physicians. Thus, the main focus of this article will be questions about how medical practices and body perceptions interact. The goal will be to connect the abandonment of bloodletting to the shift between the ‘open’ body discussed by several historians of early modern medicine, like Katharine Park and Barbara Duden, to the ‘closed’, modern body discussed by among others the anthropologist Emily Martin.^9^

## The Sources

Empirically, this article is based on materials written by Swedish medical practitioners in the nineteenth century, almost all of them doctors. Non-digitised sources include medical handbooks for the public, the two main Swedish medical journals from the period, *Eira* and *Hygiea*, and papers from Swedish medical societies (mainly *Svenska Läkaresällskapets Handlingar*), as well as lecture notes by early nineteenth-century medical students in Uppsala.^10^

Primarily, however, the study rests on both close and distant readings of 8,807 annual reports by Swedish provincial doctors, written between 1820 and 1900 and digitised in *Medicinhistoriska databasen*, run by Linköping University.^11^ All reports in this database were transcribed rather than scanned in an ambitious 1990s project funded by the Swedish government. To check whether the digitalisation process added any noteworthy distortion, 50 transcribed reports have been compared with the originals, kept at *Riksarkivet* in Stockholm. Remarkably, only two minor errors were found. The digitisation project was led by professor Roger Qvarsell at Linköping University, and personal communication with him explains the unusually high accuracy of the texts: each typed report was cross-examined by two other groups.^12^

## Methods

In a previous project, all 8,807 reports digitised in *Medicinhistoriska databasen* were read in full.^13^ Thus, this analysis begins from a thorough understanding of Swedish provincial doctors’ word use: what they discussed in their reports, which terms they used, and which medical problems mattered to them and why.

This familiarity with the source material has made it possible to choose especially interesting reports for additional analysis, and to supplement these with keyword searches. For all keyword searches discussed, other synonyms have been tested as well, but the amount of hits have been given for select words only for reasons of brevity. It should be stressed that this material is not appropriate for a full-fledged digital analysis, due to its uneven nature: more reports were digitised towards the end of the century. Although this, to some extent, has been remedied through comparison with non-digitised reports, the quantitative arguments are only meant to exemplify recurring themes. Keywords searches are used to illustrate overarching tendencies already discovered through close reading. They only support an argument mainly resting on qualitative interpretation.

## Swedish Nineteenth-Century Medicine

The nineteenth century was a period of dramatic social and political transformations. But in Swedish history, this period was characterised by ethnic and religious homogeneity, without major disruptions to everyday life through natural disasters, political revolutions or, after 1814, war. Sweden was a sparsely populated country, a fact exacerbated by the emigration of more than one million Swedes, 20 per cent of the population, towards the end of the century.^14^

The Swedish medical community was governed by a paternalistic state overseeing public health and medicine, and the medical system was characterised by firm central control, exercised through a well-organised bureaucracy with doctors acting as civil servants.^15^ In some aspects, the Swedish example mirrors the historiography of medicine in nineteenth century Europe and America, for instance the impetus cholera pandemics gave to the establishment of sanitary movements. But the Swedish medical system was characterised by a more active state involvement than most other countries.

## Provincial Doctors

Provincial doctors were state-appointed general practitioners, primarily meant to serve the poor. The category was instigated by royal ordinance in 1688, ordering the group to provide affordable health care to all who needed it as well as to report anything noteworthy from the province back to Stockholm.^16^ The system grew in fits and starts, and the entire country was not covered by provincial doctors’ districts until the late eighteenth century.^17^ Provincial doctors made up 20–30 per cent of Swedish doctors in the nineteenth century, their numbers increasing from 40 in 1805 to 299 in 1900.^18^ Apart from treating the ill, they became an increasingly important node of information for *The National Swedish Board of Health,* with which they were in continuous contact. Since their main patient group was the peasantry, their fees were fixed at a deliberately low rate, although they were allowed to supplement their income by working as private doctors to local gentry and aristocracy, and eventually as factory doctors, railway doctors and so forth. Their state income was meant to supply about half of their total annual income.^19^

There were no exact equivalents to Swedish provincial doctors internationally during the nineteenth century. However, they might be compared with similar groups in other countries, such as the Spanish *inspector provincial de sanidad,* the French *officier de santé*, and the British *Medical Officers of Health.* All Swedish provincial doctors were university educated. In Norway, there were state-appointed *distrikslege* from 1816, and in Finland, the system worked similarly to Sweden (having been the same system until 1809).^20^

The letter of instruction for Swedish provincial doctors from 1822 states that their main task was to help all ill people in the district, but they were also expected to pay attention to anything which might aid the health of the population, and anything which might impede it. Thus, they should determine all medically relevant topographical circumstances, the temperament and habits of the local population, the state of the dwellings of the peasantry, the manner in which children were raised, common folk beliefs, and so forth.^21^ Due to this almost all-encompassing task, in the early nineteenth century, most provincial doctors began their reports with a description of local circumstances, and included a myriad of details about the population. Surprisingly for the genre, the annual reports often include long descriptions of the doctors’ own practice, and not only descriptions of exceptional cases but often ordinary ones as well.

This group is often described as country doctors, but the distinction between rural and urban practice was vague, especially as most Swedish towns were small during this period.^22^ Until circa the 1860s, most provincial doctors lived in the district’s central town. Apart from running their own practice, they were increasingly expected to manage the local healthcare system on a more administrative level. They did so by overseeing midwives and vaccinators, attempting to prevent epidemic, endemic and venereal diseases, inspecting mineral wells, pharmacies and young men for the draft, as well as enacting other measurements to protect health, often by serving on local boards of sanitation. They also assisted veterinarians, performed forensic examinations and visited patients in their homes, especially during epidemics and childbirth gone wrong. Patients included members of the local gentry and bourgeoisie, as well as members of a small but growing group of industrial workers, but consisted primarily of the Swedish peasantry. Thus, a marked class difference existed between the provincial doctors and the majority of their patients. Sweden was strongly socially stratified in the nineteenth century.

The backgrounds of the provincial doctors varied, but according to biographies listing their fathers’ professions, such as captain, land owner, merchant, consul and surveyor, they were mainly from the bourgeois middle class.^23^ The state church of Sweden was Lutheran, but religion was rarely mentioned in reports, nor in memoirs or letters.^24^

Most Swedish doctors from the same generation were educated together at the universities in Uppsala or Lund. Swedish medical education was clearly regulated by the state, prescribing a set curriculum.^25^ From 1812, a double degree was required for all federal appointments, such as provincial doctor: a doctoral degree in medicine and a master’s degree in surgery.^26^ Surgical education was available only at the Karolinska Institute in Stockholm, where all Swedish medical students trained for at least a few months.^27^ Thus, the doctors’ shared outlook was shaped by their centralised education, and further supported by networks within Sweden and outside the country.

From the 1860s, the doctors’ perspectives were further homogenised as annual meetings of provincial doctors began to be held around the country, fuelled by a growing sense of shared professional identity. In 1881, the *Swedish Association of Provincial Doctors* was formed, mainly to fight for raised salaries.^28^ This association was in frequent written contact with individual provincial doctors, including those who were geographically isolated, in a lively network of knowledge exchange.

## The Provincial Doctors’ Annual Reports

Beginning in 1755, provincial doctors became required to submit annual reports describing the past year’s activities and the state of health in their district to *The National Board of Health and Welfare*.^29^ These reports were an important information-gathering tool of the Swedish state. Since the 1750s *The Office for Tabulation (Tabellverket)*, a proto-statistical government agency, pioneered demographic statistics, fuelled by mercantilist anxieties about a small population.^30^ Provincial doctors were free to design their own reports until 1851, when a template was introduced, specifically requesting information about the weather, common diseases, the doctors’ relationships with local institutions such as parish councils, health police, poor relief, prisons, vaccinators, public schools, pharmacies, midwives, spas and military personnel.^31^

These reports might be expected to have an administrative focus, but many go into extensive detail describing the doctors’ actions, cases, medical beliefs and so on. The reports also include a surprising amount of clinical notes, detailing individual treatment, especially during the first half of the century. Most are between 2 and 10 handwritten pages long.

## The Predominant Explanation for Early Nineteenth Century Bleeding: Humoral Balance

Several historians of medicine describe early nineteenth-century therapies like bloodletting as a way to restore lost balance or equilibrium. Both Charles Rosenberg and John Harley Warner state that this motive was central: Rosenberg’s oft-quoted characterisation of nineteenth-century doctors’ therapies states that ‘Equilibrium was synonymous with health, disequilibrium with disease’.^32^

This focus on humoral balance is connected with a tendency to explain bloodletting by referencing ancient texts to which an unbroken line of such practices is traced: bleeding in antiquity and in the nineteenth century is often presented as having been performed for the same reasons. For instance, Roy Porter writes that bloodletting ‘followed from humoral doctrines’ and references Galen.^33^ This is obviously correct, but bloodletting in the nineteenth century had been profoundly affected by eighteenth-century theorists, most notably by the Dutch physician Herman Boerhaave’s (1668–1738) view of the body as a mechanic system, its fluids working hydraulically by moving through ‘pipes’ under pressure, and by his Edinburgh colleague William Cullen’s (1710–90) system focusing on the irritability and sensibility of tissues and nerves.^34^ Thus, nineteenth-century bloodletting cannot be explained by looking back to ancient texts.

For Swedish nineteenth-century doctors, bleeding was almost never said to be meant to restore balance. The doctors did not discuss balancing goals. The words ‘equilibrium’ or ‘disequilibrium’ cannot be found in any of the 8,800 digitised yearly reports from provincial doctors, and the word ‘balance’ is only mentioned twice, in relation to bookkeeping.^35^ Clearly, the absence of a few synonyms is inconclusive. But other word searches also illustrate how much the humoral perspective is missing. The humoral fluids black bile and yellow bile only give four hits each, and phlegm and blood are almost never described as humours.^36^ Nor are the temperaments assumed to correspond to varying humoral balances—phlegmatic, sanguine, choleric—mentioned more than once each, and the word melancholic is used only eight times.^37^ What is lacking for the conclusion that Swedish provincial doctors’ practices were based on humoral pathology is what historian Vivian Nutton has called ‘the distinctive characteristic of humoralism’: the humours themselves.^38^ Arguments from word searches in digitised reports should be taken with a grain of salt. However, these quantitative arguments based on word searches in the digitised reports are only meant to demonstrate an observation amassed by years of close reading of Swedish provincial doctor reports, both digitised and on paper: neither balance, nor any other perceivable aspect of humoral pathology, played a decisive role in determining what provincial doctors conceived of as a medical problem or solution in the nineteenth century. The provincial doctors did not use synonyms to the humoral terms (for instance, ‘blood filled’ for sanguine).^39^ Rather, this kind of language is almost entirely lacking, both in the digitised material and in non-digitised reports held at *Riksarkivet*.

## Hydraulic, Flow-managing Medicine

Rather, Swedish provincial doctors had a hydraulic view of the body. Until circa the 1860s, many provincial doctors repeatedly stated that the bodies they treated needed to emit fluids in a steady, even flow: the body’s ability to get rid of excessive material was crucial to its health. The absence of an expected flow was a central medical problem.

Thus, they primarily bled to managed flows. Bloodletting to them was not a means to restore lost balance, to correct a quantitative relationship between amounts of fluids, but a way to increase flow speed, often by removing matter perceived as blockages. They were aiming for steady, regular movement rather than harmony.

This focus on restoring regular and calm flows is indicated by the provincial doctors’ word use. The word *jemnvikt*, a synonym of ‘equilibrium’, does not occur in any of the over 8,000 reports, whereas the word *jemn*, meaning ‘even’, was used 626 times.^40^ The word *jemn* often described even flows signalling health: an indication that a treatment had worked, or to describe what a treatment was aiming for. The speed and ease of circulation were central, not the quantity of specific fluids.

Granted, flow-managing practices were humoral in the sense that they intended to influence body fluids, but this focus on fluids was not absolute. Solid matter was also relevant if it caused blockages, and the fluids were just the medium for what was important for the provincial doctor: the movement itself.

Doctors described many kinds of blockages inside the body as important medical problems, and impeded flows were said to be harmful in several ways. Not only could they cause damage locally, in the form of congestion, but they could also make a severe impact on the whole body. The most important instance was the lack of menstruation. This was a common medical problem, understood both as symptom and cause of disease. A calm, regular, even flow was most desirable, and menstrual disorder (dysmenorrhea) or absence (amenorrhea) were frequently mentioned conditions in reports during the entire period.^41^ The provincial doctor in Luleå in 1825 described a case where a farmer’s wife sought help ‘for a variety of symptoms, clearly caused by hindered menstruation’.^42^ Writing that a woman was ‘properly menstruated’^43^ was a way for the doctor to describe her as healthy, and diseases were said to have been overcome through the return of menstruation, for instance when the doctor in the spa town Söderköping wrote that two women ‘were cured of epilepsy since the mineral water induced menstruation’.^44^

When a blockage was considered to affect the entire body’s circulation, it was called an obstruction, as in the report from Åmål in 1857 in which whooping cough was said to be caused by ‘the most stubborn obstruction’.^45^ Similarly, the doctor in Umeå in 1840 described a patient who had lived with obstruction for years and thus suffered from such severe dizziness and tinnitus that her life was threatened. But when a ‘fontanelle to the leg’ was opened up and was ‘kept open and oozing’, the dizziness and tinnitus disappeared.^46^ The artificial opening in the patient’s leg dissolved the harmful obstruction and cured the disease.

Physicians also paid attention to congestion, liquids which accumulated at a point in the body where they caused local damage. Flow-management therapies like leeches or blistering patches were used to remedy congestion by removing excessive fluids locally and were considered successful if the swelling subsided. For example, the doctor in Ljungby in 1835 considered his treatment for nerve fever successful ‘when congestion... was reduced through the evacuation of blood’.^47^

Thus, doctors treated blockages of flows within and out from the body as medical problems. Word frequencies in the digitised reports show that this focus on obstructed flows was much more common in the early 1800s than at the end of the century. For example, in relation to the number of reports, the word ‘constipation’ was seven times more common in the 1850s than in the 1890s;^48^ ‘slow/sluggish’, four times more frequent;^49^ ‘swollen’ occurred twice as often^50^ and ‘weighed down by’ was used in 36 of the reports from the 1850s but only in 4 of reports from the 1890s.^51^ Words associated with arrested and hindered circulation were thus much more frequent during the 1850s than in the 1890s.

It was also common for Swedish provincial doctors during the early nineteenth century to recount their patients’ sense of heaviness as symptoms of disease.^52^ For instance, constipation was a more medically relevant factor during the early nineteenth century than in later decades. Most often, the word ‘constipation’ (*förstoppning)* referred to difficulty expelling stool, but other types of body fluids could also be regarded as constipated.^53^ When the doctor in Västerås in 1837 described a local epidemic, he wrote that a common symptom was ‘constipation of the nose’.^54^ Swelling was also often associated with disease, as when a doctor described how ‘Swelling was accompanied by general drowsiness, headache, ... sluggish and often painful urination’.^55^ Similarly, the provincial doctor in Härjedalen in 1856 claimed cold summers to be healthiest, because in such weather the local peasant could exert himself while fishing crawfish ‘since he did not fear the blood would stiffen in his veins’.^56^ According to the doctor, peasants believed that bodily effort in hot weather made the blood’s circulation slow down, which risked creating blocked flows.

Furthermore, the meaning of the word ‘sluggish’ (*trög*) shifted during the nineteenth century. During the years 1820–70, it almost exclusively described ‘sluggish evacuation’ meaning the evacuation speed and consistency of stool.^57^ Physicians during this period often mentioned such waste as sluggish, showing their interest in the speed of the body’s internal flows. However, during the 1880s and 1890s, physicians stopped using the term in this way. Rather, they applied it to slow postal service,^58^ peasants’ reluctance towards improvements,^59^ insufficient mental activity and slow personal dispositions.^60^ Sluggishness, inertia and the lack of sufficient movement were thus described as bodily problems in the mid-1800s, but social ones at the end of the century.

Bleeding could be used to mechanically re-start a flow of blood perceived to be missing. Provincial doctors repeatedly stated that bleeding could evoke and facilitate internal flow speed. For instance, the provincial doctor in Vimmerby in 1835 was frustrated that he had to use venesection on a lame boy’s healthy limb, since ‘Bloodletting on the lame arm failed’.^61^ According to the doctor, it would have been better to bleed the lame arm, since that was where the flow was insufficient.^62^ Bloodletting could thus be a way of re-establishing blood flow within the body. The movement of the blood itself, rather than the reinstatement of lost balance, was often the main therapeutic goal. And such beliefs were shared by the public. In 1845, the doctor in Enköping stated that the local peasants ‘tend to rely on bleeding, especially if stiffness or heaviness is felt’.^63^

This view of bloodletting as a flow-managing practice was not unique to the provincial doctors, but was prevalent among many Swedish medical practitioners in the early nineteenth century. In 1804, the Swedish doctor Carl Jacob Ringblom (1776–1852) published a treatise on bloodletting in which he vigorously endorsed the use of copious bleeding to treat disease. The reason for its efficacy was its flow-managing properties. Ringblom wrote that

Phlebotomy is efficient since it reduces the amount of blood flowing in the veins, lessening the tension. This way, the blood moves more freely, and emanations can easily exit the body. Thus, the blood is cleansed, blockages removed, and drugs become more potent.^64^

In a similar manner, in 1823 the physician Albrecht Julius Segerstedt (1763–1815) wrote in a handbook in practical medicine meant for the Swedish-speaking public that indirect causes of fever included obstructions of the blood’s circulation. Thus, fever was caused by lack of proper internal movement.^65^

A third example of this hydraulic view is apparent in lecture notes taken by medical students at the university of Uppsala. During a lecture from 1804, one professor was said to have explained that bleeding could revive drowned persons because drowning hindered breathing due to congestion of fluids and ‘lack of blood circulation in the lungs’.^66^ Bloodletting could restore the blood’s flow throughout the body by ‘removing obstructions from the lungs and by triggering the circulation of the blood, which can reawaken the slumbering vitality in heart and brain’.^67^ Blood flowing out of the body mechanically restarted the internal circulation necessary for life.

To conclude: in the early nineteenth century, numerous medical problems were said to cause or be caused by blocked, slowed or missing circulation, and many therapeutic interventions tried to affect the speed of flows out of and within the body. This ties into an older tendency in Swedish conceptions of the body, observed by Anton Runesson, to associate life with movement (for instance, Runesson retells how a woman in 1715 stated that foetuses were ‘dead’ in the womb for the first four months and alive only after the quickening, when they began moving).^68^ The Hippocratic and Galenic heritage, and the dominance of humoralism, were thus less pervasive during these years than historians of medicine working on other countries have observed.

Perhaps Swedish medicine is a special case. The discrepancy compared to observations in other countries might partly have arisen from the strong influence of iatro-mechanics on Swedish medical circles in the eighteenth century. Did perchance René Descartes’ death in Stockholm in 1650 alter Swedish perceptions of the body in an idiosyncratic way? Iatro-mechanical conceptions of the body are clearly visible among such influential thinkers as Carolus Linnaeus (1707–78), the world-renowned botanist who started out as a physician and who influenced the medical faculty in Uppsala during the years of his professorship, 1741–78.^69^ Linneaus also became well acquainted with Boerhaave’s system of hydraulic medicine (as well as with Boerhaave himself, whom he knew) while studying in the Dutch Republic 1735–38.^70^ Perhaps Swedish nineteenth-century doctors were more predisposed to views of the body as a hydraulic system than doctors in other countries. But few signs indicated that the practices of Swedish doctors were dramatically different compared to their American or European peers. Rather, the fact that Swedish doctors had such clear flow-managing goals in mind with their bleeding and purging practices, indicates that there might be value in re-examining how nineteenth-century bloodletting is explained within the historiography at large.

## Prevailing Explanations for the Use of Bloodletting in the Nineteenth Century

In many popular accounts of the history of medicine, bloodletting is typically portrayed as strange, harmful and even foolish. Although professional historians of medicine usually refrain from making condemning statements outright, many historians writing about bloodletting also begin from a position of scepticism, doubting the procedure’s rationality. Roy Porter, for instance, claims about the period that ‘curing remained a subordinate consideration’ and compliments nineteenth-century doctors who dared to refrain from treating their patients because inertia was an ‘honest option’ to ‘bleeding and purging—and all the rest of the worthless mass of the pharmacopoeia’.^71^

Following this line of reasoning, bleeding is often said to have been performed for extra-medical, social reasons. One example coming from outside the history of medicine profession is the philosopher K. Codell Carter who in the book *The Decline of Therapeutic Bloodletting and the Collapse of Traditional Medicine* states that during this century, therapeutic interventions were not meant to cure patients from disease but were rather a ‘system of reinforcing social and moral norms’.^72^ This assertion is problematic. It is certainly difficult to deny that medicine is normative, but then, sickness is always deeply embedded in norms about who should take care of whom, in what circumstances people may legitimately evade their commitments, and which kinds of diseases are legitimate or not. That is hardly specific to nineteenth-century medicine. To call nineteenth-century doctors’ medical interventions socially normative is therefore not incorrect but rather misleading; it is like claiming people only eat dinner to spend time with their families.

These kinds of social explanations recur among historians of medicine as well. In *The Encyclopaedia of the History of Medicine*, Harold Cook writes that bloodletting had ‘ritualistic associations’^73^ a statement Charles Rosenberg might support, since he calls therapeutic evacuations ‘liturgical’ and bloodletting a cultural ritual.^74^ Similarly, Peter Murray Jones states that bloodletting was used to give patients ‘psychological reassurance’,^75^ an explanation also supported by Roy Porter, who stresses bedside manners as the basis of physicians’ reputations.^76^ Historians often state that doctors used bloodletting to reduce pain, calm patients and make themselves appear competent and in control. Charles Rosenberg, for example, claims that therapies must be understood to include ‘emotions and personal relationships’ and that therapeutic treatment ‘incorporates all of the cultural factors that determine belief, identity, and status’.^77^ In short, nineteenth-century bloodletting is repeatedly described by historians of medicine as an ineffective placebo.

This argument sometimes builds on the spectacular nature of blood removal. Phlebotomy is presented as a dramatic treatment which gave clear, immediate effects; the change in the sick body is impossible to question. It is repeatedly said that bleeding soothes pain and calms patients. According to Warner, bloodletting clearly demonstrated ‘the ability of the physician to alter his patient’s physiology at will’.^78^

But the use of bloodletting only to achieve immediately demonstrable physiological effects seems unlikely, given that physicians had other means for doing so during the nineteenth century. Opium, alcohol and other calming medicines were available to most doctors and gave almost instantaneous and certainly obvious effects on the well-being of the patient. Pain and excessive excitement were easily reduced through such means. If doctors had been focusing on their own prestige, it would have been more strategic to stress the superiority of treatments which only they could provide, like opium, whereas bloodletting could be performed by just about anyone.

Assuming that doctors’ bled patients to make themselves seem more competent also makes the abandonment of bloodletting difficult to understand. Does not the physician’s need to appear reliable, competent and in control remain constant? Explaining bloodletting as a way for doctors to provide reassurance does not explain its abandonment, since this has always been part of doctors’ responsibilities.

Bleeding was certainly used for social reasons to some extent. But even as late as the middle of the nineteenth century, many doctors also saw it as an effective, flow-managing therapy. And since bloodletting was used for flow-management, one important motivation for doctors to stop bleeding their patients was a little-discussed shift during the nineteenth century: the transition between flow-managing and boundary-protecting medicine, and the corresponding change from an open, flowing body to a closed, autonomous one.

## The Open Body Closed

Many historians of the body such as Gianna Pomata, Katharine Park and Barbara Duden have described the shift in body conceptions from an open to a closed body within Western medicine.^79^ Seeing a body as ‘open’ means understanding it as malleable and related to its surrounding milieu, the seasons, other people, the stars and so on, whereas a closed body is clearly separate from its environment.^80^ The transition between these two conceptions of the relationship between bodies and their environments is generally said to have taken place during the Enlightenment or even the Renaissance; the open body has been called ‘pre-modern’.^81^ But as shown, Swedish provincial doctors engaged with a body constituted by fluidity and mobility until at least the 1860s. The reasons for the shift to perceiving bodies as closed and autonomous are hard to surmise through provincial doctors’ reports. But it is clear that within Swedish medicine, flow-managing medicine was not abandoned until the mid-nineteenth century.

## Bloodletting Abandoned in Sweden

From the mid-nineteenth century onwards, bleeding became more and more rare in the Swedish context, and began being described as something harmful. It was said to impair human health, age people prematurely and sometimes even cause their death.^82^ Around 1880, for example, the doctor in Gislaved stated that in his district ‘the direct cause of Chlorosis is the recurring habit of opening veins’.^83^ His colleague Waldemarsvik claimed that many diseases stemmed from the tendency among peasants to ‘bleed themselves for every little thing’.^84^ References to bloodletting were now often used to illustrate the malignancy of quacks’ treatments, and to be described as using bleeding now suggested one was an illegitimate medical practitioner.^85^ Although there are examples of provincial doctors applying leeches therapeutically in 1881, and one provincial doctor paid a midwife to bleed patients as late as 1895, leeching was only a common practice among them until the early 1860s.^86^ In 1862, one provincial doctor wrote that leeches ‘are not harmful but beneficial’^87^ which shows that he perceived their use as questionable. Beginning in the late 1860s, bloodletting was increasingly said to be a practice of quacks and peasants rather than of doctors, and blood removal was said to cause diseases, not cure them. Doctors abandoned bloodletting not by openly evaluating the strengths and weaknesses of the practice, but silently: in the reports, doctors went from describing themselves prescribing bleeding to saying that this was a practice only performed by the ignorant. To use bloodletting was now presented as evidence of foolishness: ‘There is a great deal of superstition and tomfoolery concerning the treatment of illness [among peasants]... Their most common cure is phlebotomy’.^88^

From circa the 1860s onwards, bloodletting tended to be described as weakening, as a dangerous way to intentionally make bodies lose blood they desperately needed. One doctor wrote that a local quack had ‘bled Jansson’s wife so thoroughly that she fainted from blood loss, and then became ill for a long time’.^89^ In the same way, the doctor in Filipstad explained that frequent phlebotomy by a quack had greatly enfeebled his patient.^90^ Although doctors were aware of this danger even in the early nineteenth century, the discussions about the risks of bloodloss became much more prevalent. Provincial doctors thus began taking it upon themselves to prevent bloodletting. In 1860, doctor Malmberg in Ramsberg wrote, ‘I am very happy to say that during this year, venesections, at least in the area surrounding my station, have been more rarely used than previous years, especially after I myself have begun to sternly address those people, who practice this despicable art’.^91^ Doctor Malmberg had himself prescribed therapeutic bloodletting only two years earlier.^92^

But why did the doctors stop bleeding? Very few nineteenth-century physicians openly stated that bloodletting was harmful or ineffective. An examination of the indexes for all nineteenth-century issues of the two main Swedish medical journals, *Hygiea* and *Eira*, as well as the papers of the main Swedish medical association, *Svenska Läkaresällskapet,* reveals no discussions about the value and efficiency of bloodletting.^93^ Internationally, historians of medicine have gone through medical journals and the proceedings of medical societies from several countries without finding much discussion about the worth of bloodletting.^94^ There are two main exceptions to this rule: occasions when the practice of bloodletting were openly discussed as potentially harmful in the nineteenth century. One discussion resulted from the French doctor Pierre-Charles-Alexandre Louis’ statistical studies in the 1820s and 1830s, and one public debate took place between two Edinburgh professors in the 1850s. Several historians who argue that bloodletting, in the nineteenth century, was abandoned because it had been proven ineffectual on empirical grounds, support this assertion almost entirely on the basis of Louis’ study *Recherches sur les effets de la saignée*, first published in 1828.^95^ Here Louis, who later would become a key figure in the establishment of medical statistics in France, investigated whether bloodletting improved or worsened patients’ conditions and rates of recovery in certain inflammatory diseases. He found that bloodletting had limited influence. But his study did not conclusively prove it to be ineffective. Louis himself concluded that ‘bloodletting, notwithstanding its influence is limited, should not be neglected in inflammations’.^96^ He even regretted that others interpreted his study as questioning the value of venesection. In the introduction to the American translation of one of his books, Louis expressed sadness that ‘some in consequence of prejudices, difficult of explanation, declared that I rejected bloodletting in the treatment of cases of inflammation, although I show the necessity of having recourse to it’.^97^ Louis’ investigation thus used statistical methods to show that bloodletting was sometimes less effective than previously thought, but it did not lead him to proclaim the need to abandon it: instead, he emphasised that phlebotomy could be an important therapy which should not be neglected.

Few doctors were openly critical of bloodletting as it was being abandoned. As John Harley Warner has shown, the most important of these scarce dissenters was John Hughes Bennett. He was a professor of medicine at the University of Edinburgh who, during the 1850s, was involved in a public discussion concerning the value of bloodletting with William Pulteney Alison, another professor of medicine at the same university. Warner calls this ‘the Edinburgh bloodletting controversy’, and highlights some startling circumstances.^98^ Not only was this one of very few substantial debates about the value of bloodletting; almost all participants rallied to Alison’s side and defended the practice against Bennett’s criticism. Thus, it would be difficult to argue that Bennett disproved bloodletting.

Historians of medicine have further explained the abandonment of bloodletting by discussing social reasons. One such argument focuses on economy: doctors are said to have ceased bleeding and purging since such draconian treatments were a liability compared to homeopathic treatments in the open competition for well-to-do patients. Therefore, doctors’ therapies were ‘modified to fit economic realities’.^99^ Others argue that bloodletting was under severe attack from homeopaths and Thomsonites.^100^ However, this does not explain why bloodletting disappeared at the same pace in countries where alternative medicine did not have much influence during the nineteenth century, as in Sweden. Furthermore, it seems strange that opinions expressed by homeopaths would have succeeded in undermining bloodletting, a practice which at this time served as a symbol for orthodox medicine. Orthodox medicine is constantly being criticised by alternative medicine to little or no effect, and as several historians of medicine have demonstrated, the medical marketplace had already widened remarkably in the eighteenth century in several countries.^101^ Why would objections raised by alternative medicine be able to undermine the practice of bloodletting, when criticism of other medical practices has had little effect? Alternative medicine generally defines itself as that which orthodox medicine is not. Thus, orthodox medicine rarely heeds criticism from such perspectives.

The argument that these attacks had an indirect effect by changing patients’ perceptions of correct treatment is also problematic. The assumption is that altered demand from patients led to the abandonment of bloodletting: patients refused to be bled, and doctors stopped prescribing bleeding. However, the reluctance of patients does not explain why bloodletting was abandoned; it only removes the question one step further from the practice without establishing why patients did not want to be bled anymore.

Codell Carter proposes that bleeding became rarer since doctors’ clientele changed; whereas doctors at the beginning of the century were treating mainly aristocrats, who were honour bound to endure bleeding, during the mid-1800s, doctors began treating poorer, weaker people who did not fare well with blood loss.^102^ This is a variant of nineteenth-century doctors’ own explanations, but does not explain why phlebotomy disappeared at the same rate among Swedish provincial doctors, whose clientele remained unchanged socially throughout the nineteenth century (as this group had treated poor patients for centuries).^103^

## The Closed Body Emerges

The silences surrounding the abandonment of bloodletting make it difficult to make a definitive statement about why it stopped being seen as effective. All the reasons discussed above probably had some influence on the abandonment of bloodletting, which certainly was a slow, complex and multicausal process. But the transition between the open to the closed body presents a new, not previously discussed rationale for this change, which should be taken into account. Although the empirical material presented in this article does not provide enough insight into why and how this process changed, historians working on materials from other countries have noticed the same shift, and their work might provide a clue to ways to begin examining nineteenth-century orthodox medicine from the perspective of body perceptions.

Scholars working on conceptions of medicine in the USA, such as Susan Sontag and the anthropologist Emily Martin, argue that the late nineteenth century was characterised by medicine which aimed to protect the boundaries between bodies and their environments. Transitioning from flow-management into a new kind of boundary-protecting medicine during the last decades of the century was coupled with a transition between two conceptions of how the body related to its environment; open and engaged in constant interaction, or closed and autonomous. This shift can be seen as one of several reasons why therapeutic bleeding was abandoned, because purging and bloodletting assumed a body in which the circulation of fluids was central to health. In order for therapeutic removal of bodily fluids to be rational, it must be understood as removing excesses or obstacles to the processes which characterised good health: even and predictable circulation. But in the late nineteenth century, the removal of body fluids began to be understood in a completely different way: as a loss.

One sign that doctors began conceiving the body as a discrete, separate entity in the 1860s and onward is that bodies were increasingly described as economic units, which should avoid losses. Metaphors relating to saving and wastefulness became common.^104^

In *Illness as Metaphor*, Susan Sontag suggests that late nineteenth-century perceptions of disease

… echo the attitudes of early capitalist accumulation. One has a limited amount of energy, which must be properly spent... Energy, life savings, can be depleted, can run out or be used up, through reckless expenditure. The body will start ‘consuming’ itself, the patient will ‘waste away’.^105^

This closed body ought to be preserved, since a lack of energy or vitality was its central medical problem. Removing body fluids, in this view, became wasteful. Emily Martin writes about perceptions among American doctors during the end of the nineteenth century that ‘the shift from the body as an intake-outgo system to the body as a small business trying to spend, save or balance its accounts is a radical one’.^106^ According to Martin, the difference between metaphors which liken the body to something which interacts with its environment and those that explain excretions as losses is the key to understanding late nineteenth-century medicine.^107^ As an example, Martin takes an American biologist who in the late nineteenth century discussed cell activity in economic terms, as accumulative and degradative processes.^108^ Martin also points out that in medical handbooks, menstruation was reconfigured from purification to pathology: menstruation was now presented, in the words of a nineteenth-century doctor, as a way in which women were ‘periodically wounded’.^109^ In addition, she argues that this new anxiety led some doctors to redefine menopause as a positive change, since it meant that menstruation—now described as a harmful recurring blood loss—came to an end.^110^ Another example of this new medical anxiety over loss of body fluids is the way some doctors began describing the body as a closed system of energies and liquids which had to be managed.

The Swedish historian of ideas Maja Bondestam describes how claims that different ages demand varying amounts of a limited quantity of energy or vitality became common around the mid-1800s.^111^ According to historian Athena Vrettos, the formulation of the energy principle (the first principle of thermodynamics) in the 1840s made economic theories of body and mind popular after the middle of the century. The energy principle states that energy cannot be created or destroyed, but only converted from one form to another, and was used analogously in a wide range of areas, most prominently within medicine.^112^ For instance, the most outspoken opponent of bloodletting during the nineteenth century, John Hughes Bennett, opposed the practice precisely because it meant a loss for the body’s ‘economy’.^113^

During the same decades, a debate about the dangers of masturbation (a worry expressed for centuries) became very widespread all over the United States and Europe, including in Sweden, and the topic was extensively discussed in medical journals throughout the second half of the nineteenth century.^114^

A principal argument against (male) masturbation was precisely that the vital force was wasted through unnecessary expenditure of sperm. The body was understood to have a limited amount of energy which materialised in various secretions: semen was believed to be intimately connected with the forces of life. One handbook claimed that it contained vitality in its most concentrated form; other texts took as proof the fatigue which overtook men after intercourse.^115^ The loss of bodily fluids was the main medical problem. Ben Baker-Benfield has called this theory ‘the spermatic economy’, and Clas Ekenstam, who has analysed the Swedish debate about masturbation, emphasises how common it was to intermingle economic and sexual terms.^116^ ‘The body’, Ekenstam writes, ‘was seen as a production system with only a restricted amount of material at its disposal…. The limited supply of semen meant any waste had immense consequences.... Those who lacked vitality were often asked to completely abstain from intercourse’.^117^ Among such people were those who had open wounds and who were therefore already losing body fluids.^118^ Ejaculation of semen became a kind of drainage of vital energy.^119^ Swedish provincial doctors also associated masturbation with weakness from mid-century, as when the doctor in Grästorp wrote in 1891 that neurasthenia was caused by ‘masturbation among children and adolescents’.^120^

In a similar way, a Swedish dissertation claimed that it was wasteful for men to shave their beards, since a clean-shaved body demanded energy in order to recreate lost hair. Wasting semen was also said to result in hair loss since bodily fluids otherwise designated for hair were used for recreating seminal fluid.^121^ It was now considered irresponsible, wasteful and harmful to allow bodily fluids to flow out from the body if it could be avoided. Practically all body fluids were now assumed to be most needed inside the body. There are thus multiple examples of how the loss of body fluids was presented as dangerous in the late 1800s. Most interesting for this paper, however, is perceptions of blood removal. Swedish sociologist Boel Berner has shown how blood transfusions between humans, also a topic of medical interest for centuries, were attempted with increasing frequency in the 1870s, both in Sweden and beyond. One major problem was that it was considered unethical to remove blood from human donors, leading doctors to attempt transfusions with blood from lambs.^122^ This method was said to be preferable partly because it could be difficult to find people willing to ‘give up their blood to another’ since blood donation was too invasive a procedure to put healthy people through.^123^ This view suggests a new perception of what could harm a body, only a few years after doctors routinely used bloodletting as therapy. That the practice of removing blood, which for millennia had been considered beneficial to health, now was declared as potentially harmful, suggests a fundamental change in the perception of how a body worked.

The 1870s was also the decade when the first article on ‘surgical blood-saving methods’ appeared in a Swedish medical journal. The article, published in *Hygiea*, described it as problematic that the removal of tumours tended to cause extensive bleeding, but described methods which allowed surgery while only losing a single ‘teaspoon of blood’.^124^ Such success was contrasted to an operation using conventional methods, after which ‘waxy skin colour, small, weak pulse and laborious breath signalled anaemia’.^125^ It is interesting that a weak pulse was taken as a sign the patient had lost too much blood, because within flow-managing medicine, a weak pulse could indicate lack of circulation and thus signal the need for bloodletting. For example, when in 1845 the provincial doctor Varenius treated a farmer who had been attacked with an axe to the head, his first order of business was bleeding in order to stimulate circulation: ‘[the farmer had] an almost negligible pulse. Therefore, I immediately opened his veins. Once the pulse had risen and was free, I began to investigate the injury’.^126^ The difference between treating the open and the closed body is that for the closed body, the total blood volume was important, whereas for the open body, the calm, regular flow of bodily fluids was the principal sign of health.

## Conclusion

Based on Swedish provincial doctors’ practices, the common assertions by historians of medicine that early nineteenth-century doctors bled their patients either to restore lost humoral balance, or for extra-medical, social reasons, have been put into question.

Social reasons for medicine always exist to a certain extent—doctors need to make a living, present themselves as competent. The efficiency of placebo effects is well documented. However, within the flow-managing medicine framework, bloodletting *was* medically efficient: blocked flows caused stagnation which harmed health, while bloodletting removed blockages and improved flow speed.

Of course, the hydraulic and the humoralist views do not need to be mutually exclusive. It might well be argued that the elimination of blockages could be constructed as a way of restoring balance (even if the latter goal was so self-evident that it did not need to be stated) and that the doctors’ fascination with loss and retention was not a radical departure from the Galenic inheritance (excretions and retentions being one of the six non-naturals). However, some reference to the quantity of fluids removed should be present. Otherwise, ‘balance’ is only being used as a vague synonym for health.

The closed body did not need to be bled. Rather, bleeding hurt and weakened it. This difference, and the dissimilarities between the open and closed bodies, can provide a supplemental explanation for why therapies stimulating flows within and out from bodies disappeared. If internal circulation no longer needed to be maintained, bloodletting became ineffective and meaningless.

Of course, the rapid success of bacteriology from the 1870s onwards was a driving force for the growth of scientific medicine. But bacteriology, which states that disease is caused by harmful agents penetrating the body’s defences, also fits very well with beliefs about how the closed, autonomous body interacted with its environment—beliefs which, as has been shown, pre-dated it.

Body perceptions are tacit assumptions underlying medical practitioners’ actions and beliefs. These kinds of assumptions operate at a level which is often considered so basic that it is rarely discussed openly. Bleeding began to be seen as hurting bodies rather than saving them; the logic behind it became perplexing, even incomprehensible. This transition was not the result of open statements or of choosing sides; rather, an earlier way of thinking and acting became unintelligible.

## Footnotes

1 Phlebotomy (from the Greek ‘phlebos’, meaning ‘vein’ and ‘temnein’, meaning ‘to cut’) is a synonym to bloodletting and to venesection. It means using a sharp object to open a patient’s vein to drain blood. This procedure was often applied to a forearm, and was generally intended to remove blood from the entire body. In contrast, leeching—the application of medical leeches (*Hirudo medicinalis*) on for instance the gums or in the throat—was usually meant to remedy local swelling.

2 Roy T. Sawyer, ‘History of the Leech Trade in Ireland, 1750–1915: Microcosm of a Global Commodity’, *Medical History*, 2013, 57, 420–41, 421.

3 Unsigned introduction, *The Lancet,* 1823, 1, 1–2.

4 John Harley Warner, *The Therapeutic Perspective: Medical Practice, Knowledge, and Identity in America, 1820–1885* (Princeton: Princeton University Press, 1997), 5.

5 Charles E. Rosenberg, ‘The Therapeutic Revolution: Medicine, Meaning, and Social Change in Nineteenth-Century America’, *Perspectives in Biology and Medicine,* 20, 1977, 4, 485–506, 485.

6 Shigehisa Kuriyama, ‘Interpreting the History of Bloodletting’, *Journal of the History of Medicine and Allied Sciences,* 1995, 50, 11–46, 11. Kuriyama explains bloodletting through investigating ancient texts and does not return to the question of why it was abandoned in the nineteenth century.

7 Rosenberg, *Therapeutic Revolution;* Warner, *Therapeutic Perspective*; David Wootton, *Bad Medicine: Doctors Doing Harm Since Hippocrates* (Oxford: Oxford University Press, 2007); K. Codell Carter, *The Decline of Therapeutic Bloodletting and the Collapse of Traditional Medicine* (New Brunswick, NJ: Transaction Publishers, 2012).

8 Warner, *Therapeutic Perspective*, 4.

9 Emily Martin, The *Woman in the Body: A Cultural Analysis of Reproduction* (New York, NY: Beacon Press, 2001). For descriptions of the premodern fluid and open body, see Barbara Duden, *The Woman Beneath the Skin* (Cambridge, MA: Harvard University Press, 1991); Gianna Pomata, *Contracting a Cure: Healers and the Law in Early Modern Bologna* (Baltimore, MD: Johns Hopkins University Press, 1998); Katharine Park, ‘Was There a Renaissance Body?’, in W. Kaiser and M. Rocke, eds, *The Italian Renaissance in the Twentieth Century*, Tatti Studies 19 (Florence: Olschki, 2002), 21–35.

10 The journals were founded in 1839 and 1877, respectively, and all issues of *Hygiea: Medicinsk och Farmaceutisk Månadstidskrift* between the years 1839–1900, and of *Eira: Tidskrift för Helso- och Sjukvård* between the years 1877–1900 have been investigated. The search for other popular publications has relied on digitised collections of the *Internet Archive* in Swedish, and the non-digitised collections at *Kungliga biblioteket* and *Riksarkivet* in Stockholm and *Carolina Rediviva* in Uppsala.

11
*Medicinhistoriska databasen,* https://ep.liu.se/databases/medhist/

12 Conversation between Roger Qvarsell and the author on August 28, 2017.

13 Annelie Drakman, *När kroppen slöt sig och blev fast: Varför åderlåtning, miasmateori och kklimatmedicin övergavs vid 1800-talets mitt* (Uppsala: Acta Universitatis Upsaliensis, 2018)

14 Lennart Pehrson, *Den nya världen: utvandringen till Amerika* (Stockholm: Bonniers, 2014); In 1800, the population was 2.3 million; in 1900, 5.2 million. As stated by *Statistics Sweden,* Statistiska Centralbyrån, ‘Sveriges folkmängd från 1749 och fram till idag’, published October 27, 2017, https://www.scb.se/hitta-statistik/artiklar/2017/sveriges-folkmangd-fran-1749-och-fram-till-idag. Accessed May 20, 2022.

15 Karin Johannisson, ‘The People’s Health: Public Health Policies in Sweden’, *Clio Medica*, 1994, 26, 165–82, 168.

16 Göran Gerner, *Svenska Provinsialläkarföreningen 1880–1972* (Stockholm: SPLF, 1975), 9–11, 9.

17
*Ibid*.

18 Anna Furhoff, ‘Svensk allmänmedicin—när praktiken fick en teori’, in Nils O. Sjöstrand, ed., *Ett sekel med läkaren i fokus: Läkarförbundet 1903–2003* (Stockholm: Läkarförbundet, 2003), 251; Motzi Eklöf, *Läkarens ethos: Studier i den svenska läkarkårens identiteter, intressen och ideal 1890–1960* (Linköping: Linköping University Press, 2000), 199.

19 Folder named ‘Afskrifter’, *Svenska provinsialläkarföreningens arkiv.* Unpublished, from the archive of the Swedish provincial doctors’ association, *Swedish Medical Association*, Stockholm.

20 Between 1814 and 1905, Sweden was in a union with Norway, but the systems of provincial doctors were run separately. See Aina Schiøtz, *Distriktslegen—institusjonen som forsvant: Det offentlige legevesen 1900–1984* (Oslo: Senter for helseadministrasjon, 2000); Joseph Barona and Steven Cherry, eds, *Health and Medicine in Rural Europe (1850–1945*) (Valencia: Publicacions de la Universitat de València, 2005), 106, 43, 74; Otto E. A. Hjelt, *Svenska och finska medicinalverkets historia 1663–1812: Del II* (Helsingfors: Helsingfors centraltryckeri, 1891), 60–73; Saara-Maija Kontturi, ‘Läkande, botande och bilden av den kompetenta medicinaren i de finländska läkarnas årsberättelser 1769–1856’, *Historisk Tidskrift för Finland*, 2021, 105, 497–516; John Cule, *A Doctor for the People. 2000 Years of General Practice in Britain* (London: Springer, 1980); Sten Lindroth, *Svensk lärdomhistoria: Frihetstiden* (Stockholm, 1978), 421; Hilding Bergstrand, ‘Läkarkåren och provinsialläkarväsendet’, in Hilding Bergstrand, ed., *Svenska läkaresällskapet 150 år: Dess tillkomst och utveckling: En återblick* (Lund: Svenska läkaresällskapet, 1958), 60, 120–24; Carl Dahlborg, *Svenska provinsialläkarföreningens historia 1881–1905* (Stockholm: Svenska tryckeriaktiebolaget, 1905), 14–15.

21 Dahlborg, *Svenska provinsialläkarföreningens historia*, 14.

22 Ylva Sjöstrand, ‘Modernitet och traditionalism i svenska provinsialläkarberättelser 1851–1874’, *Svensk Medicinhistorisk Tidskrift*, 2003, 7, 139–61; Anna Prestjan, ‘State’s Medical Experts in Local Practice: Provincial Doctors View of Themselves as Public Health Promoters: An Example from the Swedish Countryside, 1880–1920’ *Hygiea Internationalis*, 2010, 9, 223–46; Peter Nilsson, ‘Folkhälsan i Östskåne under mitten av 1800-talet speglad via en provinsialläkares arbete’, *Svensk Medicinhistorisk Tidskrift*, 2002, 6, 121–27; Sören Edvinsson, ‘A Country Doctor: Health Care in a Mid-Nineteenth–Century Swedish Remote Area’, in J. T. H. Connor and Stephan Curtis, eds, *Medicine in the Remote and Rural North, 1800–2000* (London: Routledge, 2011), 93–114.

23 A. J. Bruzelius, *Sveriges Läkare-historia Ifrån Konung Gustaf den I:s till närvarande tid: Tredje följden. Del I; Läkarmatrikel* (Stockholm: Svenska läkaresällskapet, 1895).

24 For memoirs, see, for instance, Einar Wallqvist, ‘*Kan doktorn komma?’: läkarhistorier från lappmarken* (Stockholm: Bonnier, 1935). For letters, see the private archives of provincial doctors, for instance, *Förste provinsialläkarens i Jämtlands län arkiv 1860–1981*, Landsarkivet Östersund. The word ‘God’ (‘Gud’ in Swedish) is mentioned 64 times in the digitised reports, but more than half of the time, the word is used when the doctor quotes patients, often to illustrate the fatalism of peasants.

25 Dahlborg, *Svenska provinsialläkarföreningens historia*, 20–30.

26 Gunnar Broberg, ‘Liten svensk medicinhistoria’, in Gunnar Broberg, ed., *Til at stwdera läkedom: Tio studier i svensk medicinhistoria.* (Stockholm: Sekel, 2008), 9–50.

27 Bergstrand, ‘Läkarkåren och provinsialläkarväsendet’, 109.

28 Folder named ‘Afskrifter’, *Svenska provinsialläkarföreningens arkiv.* Unpublished, from the archive of the Swedish provincial doctors’ association, *Swedish Medical Association*, Stockholm.

29 The National Board of Health and Welfare operated as Collegium Medicum 1663–1813, Sundhetskollegium 1813–1877 Medicinalstyrelsen 1878–1967, and Socialstyrelsen 1968–.

30 Karin Johannisson, *Kroppens tunna skal: Sex essäer om kropp, historia och kultur* (Stockholm: Norstedts, 1997), 44–50.

31 Bengt Dahlin and Jan Kuuse, *Från provinsialläkare till primärvård: En historisk exposé med exempel från Mittenälvsborg i Västra Götalandsregionen* (Lerum: Bengt Dahlin, 2010), 54.

32 Rosenberg, ‘Therapeutic Revolution’, 485. See also Warner, *Therapeutic Perspective.*

33 Roy Porter, *Blood and Guts: A Short History of Medicine* (London: Penguin, 2003), 115. See also Kuriyama, ‘Interpreting the History of Bloodletting’.

34 Leonard G. Wilson, ‘Fevers’, in R. Porter and W. H. Bynum, eds, *Companion Encyclopedia of the History of Medicine* (London: Routledge, 2004), 397–400.

35
*Ekvilibrium* and *balans*, respectively. All translations from Swedish are my own.

36
*Svart galla* and *gul galla*, respectively. *Slem* (phlegm) returns 151 hits and *blod* (blood) 339 hits, but since these terms are also used in ways unrelated to humoralism, these numbers are misleading.

37 In Swedish *flegmatisk*, *sangvinisk*, *kolerisk* and *melankolisk*. Of the eight hits for *melankolisk*, all but one is used in a non-humoral way.

38 Vivian Nutton, ‘Humoralism’, in Porter and Bynum, *Companion Encyclopedia*, 282.

39 The following synonyms have been used for key word searches in the database, all resulting in a negligible amount of hits which do not counter the argument presented in this article: *blodfull* (‘blood filled’), *mjältsjuk* (‘sickness of the spleen’), *eukrasia/is, dyscracia/is* (terms used in ancient humoral medicine) *vätskelära* (‘humoralism’), *vätska/vätskor* (‘fluid/s’), *pletorisk* (‘plethoric’).

40 Spelling variations have been accounted for concerning all search words in the database. *Jämvikt*, *jämnvikt*, *jemvikt* and *jemnvikt* yield no hits. *Jämn* yields 78 hits, the same word spelled *jemn* gives 548 hits. See annual reports from provincial doctors. All quoted reports can be found through searches in *Medicinhistoriska databasen* using the doctors’ last name, their location and the year of the report (last visited August 24, 2024). For reports where the word ‘jemn’ is used, see for instance doctor Elmlund in Borås 1833; doctor Fornander in Jämtland 1851; doctor Selldén in Söderfors in 1881.

41 For dysmenorrhea as a diagnosis, see doctor Aurell in Vingåker 1844; doctor Strandberg in Undersåker 1856; doctor Rydberg in Ronneby 1858; doctor Kolmodin in Slite 1879; doctor Wessén in Hudiksvall 1889.

42 Doctor Swedmark in Kisa 1837. See also doctor Forling in Västerviks stad 1879.

43 Doctor Hanström in Vimmerby 1838; see also doctor Segerstedt in Åmål 1848.

44 Doctor Lagberg in Söderköping 1825.

45 Doctor Segerstedt in Åmål 1857.

46 Doctor Hay in Umeå 1840.

47 Doctor Billengren in Ljungby 1835.

48 Since there are 2632 reports digitised from the 1890s, nearly twice as many as the 1410 from the 1850s, the same number of hits from the two decades would mean that the frequency had been reduced by half. The term *förstoppning* (‘constipation’) yields the following number of hits: 1890s, 13; 1880s, 29; 1870s, 6; 1860s, 31; 1850s, 54; 1840s, 24; 1830s, 8; 1820s, 4.

49 Search word *trög* (‘slow/sluggish’), frequency: 1890s, 8; 1880s, 15; 1870s, 7; 1860s, 12; 1850s, 20; 1840s, 18; 1830s, 4; 1820s, 2.

50 Search word *svullen* (‘swollen’), frequency: 1890s, 12; 1880s, 18; 1870s, 3; 1860s, 13; 1850s, 13; 1840s, 7; 1830s, 3; 1820s, 5.

51 Search word *tyngd* (‘heaviness, weighed down by’) frequency: 1890s, 4; 1880s, 13; 1870s, 4; 1860s, 13; 1850s, 36; 1840s, 36; 1830s, 12; 1820s, 9.

52 The search word *tyngd* gives 127 hits. During the early nineteenth century, it was always used to describe symptoms, but during the end of the century, it was also used metaphorically, for instance, describing families burdened by many children. See Doctor Wejlander in Malmö 1820; Doctor Eneroth in Jönköping 1823; Doctor Waldenström in Luleå 1823; Doctor Boustedt in Orust 1840; Doctor Peters in Klippan 1900.

53 The search word *förstoppning* (‘constipation’) gives 168 hits. See, for instance, doctor Hanström in Vimmerby 1835; doctor Witt in Trosa Stad 1844; doctor Hallström in Lycksele 1844; doctor Cederschjöld in Nora 1849; doctor Hanström in Vimmerby 1835.

54 Doctor Ringenson in Västerås 1837.

55 Doctor Beronius in Falu Gruvor 1851.

56 Doctor Ellmin in Härjedalen 1856.

57 Doctor Boustedt in Orust 1840; doctor Swedenborg in Sölvesborg 1841; doctor Ekman in Kalmar 1840.

58 Doctor Brattström in Lenhovda 1897.

59 Doctor Lundh in Åsele 1897; doctor Arnell in Rejmyre 1900; doctors Geete/Andréen in Borås 1894.

60 Doctor Gullström in Älvdalen 1894; doctor Tham in Dannemora 1890; doctor Mitander in Föllinge 1894. Provincial doctor Svenonius in Hede 1889 made the boldest claim, claiming that ‘The peasants are sluggish in everything’.

61 Doctor Hanström in Vimmerby 1835.

62
*Ibid*.

63 Doctor Martin in Enköping 1845.

64 Carl Jacob Ringblom, *Afhandling om åderlåtning: Dess nytta och werkningar, samt om blodiglar och spanska flugor, deras nytta wid förefallande sjukdomar, och sättet at applicera dem. Utgifwen i sammandrag til swenska allmogens nytta* (Stockholm: Jonas Lötström, 1804), 18.

65 Albrecht Julius Segerstedt, *Om Medicinen: Lärobok uti Medicinen innehållande kort underrättelse om de allmännaste sjukdomars kännetecken, skötsel och enklaste botningssätt* (Stockholm: Segerstedt, 1823), 4.

66
*Föreläsningar öfver Pathologica och Therapia Speciale* (Upsala: 1804), 74–75. Unpublished notes, *Carolina Rediviva*, Uppsala University Library.

67
*Ibid.*, 74.

68 Anton Runesson, *Blod, kött och tårar. Kroppslig erfarenhet i Sverige, ca 1600–1750* (Stockholm: Historiska institutionen, 2021), 55.

69 David Dunér, ‘Maskinen människa: iatromekaniken i Sverige’, Gunnar Broberg, ed., *Til at stwdera läkedom: Tio studier i svensk medicinhistoria,* (Stockholm: Sekel, 2008), 111–49.

70 Gunnar Broberg, *Carl Linnaeus* (Stockholm: Swedish Institute, 2006), 88.

71 Roy Porter, *The Greatest Benefit to Mankind* (New York, NY: W. W. Norton, 1999), 680.

72 Codell Carter, *Decline of Therapeutic Bloodletting*, 6.

73 Harold J. Cook, ‘Physical Methods’, Porter and Bynum, *Companion Encyclopedia*, 940.

74 Rosenberg, ‘Therapeutic Revolution’, 492.

75 Peter Murray Jones, *Medieval Medical Miniatures* (London: British Library, 1984), 121.

76 Porter, *Greatest Benefit to Mankind*, 256.

77 Rosenberg, ‘Therapeutic Revolution’, 485–86.

78 Warner, *Therapeutic Perspective*, 92.

79 Gianna Pomata, ‘Innate Heat, Radical Moisture and Generation’, in N. Hopwood, R. Flemming and L. Kassell eds, *Reproduction: Antiquity to the Present Day* (Cambridge: Cambridge University Press, 2018), 195–208; Duden, *The Woman Beneath the Skin*; Park, ‘Was There a Renaissance Body?’

80 Runesson, *Blod, kött och tårar*.

81 Duden, *The Woman Beneath the Skin*.

82 N. P. Hamnberg, ‘Om Marienbad, dess helsokällor och bad’, *Hygiea*, 1870, 32, 1–5.

83 Doctor Norstedt in Waldermarsviks distrikt 1880.

84 Doctor Svensson in Gislaved 1879. See also doctor J. A. Leffler in Våla 1864.

85 Doctor Carlgren in Skog 1865; doctor Zetterstrand in Malmköping 1858. An example of a doctor saying bloodletting was the practice of a quack can be found in doctor Drake in Sigtuna 1878.

86 For the decline, see Drakman, *När kroppen slöt sig och blev fast.* Doctor Selldén in Söderfors 1881; doctor Johansson in Rejmyre 1895. See also Warner, *Therapeutic Perspective*, 115.

87 Doctor Westerberg in Sundsvall 1862.

88 Doctor Carlgren in Skog 1863; doctor Ramnäs in Westerås 1868; doctor Pallin in Filipstad 1856.

89 Doctor Selldén in Söderfors 1881.

90 Doctor Pallin in Filipstad 1856.

91 Doctor Malmberg in Ramsberg 1860.

92 Doctor Malmberg in Ramsberg 1858. To treat meningitis, doctor Malmberg used ‘bloodletting and cupping in the neck’.

93 There are several articles about bloodletting but none which question or evaluate the worth of the practice. See, for instance, J. Levertin, ‘Om behandling af Pneumonie hos äldre personer med åderlåtningar och Tartar’, *Hygiea*, 1842, 7, 40–42.

94 Codell Carter, *Decline of Therapeutic Bloodletting*, 3.

95 Irving Loudon, ed., *Western Medicine: An Illustrated History* (Oxford: Oxford University Press, 2005), 94; Erwin Heinz Ackerknecht, *A Short History of Medicine* (Baltimore, MD: Johns Hopkins University Press, 1982), 134.

96 Wootton, *Bad Medicine*, 171.

97 Pierre-Charles-Alexandre Louis, *Researches on the Effects of Bloodletting in Some Inflammatory Diseases, and on the Influence of Tartarized Antimony and Vesication in Pneumonitis* (Boston, MA: Hilliard, 1835), xxxi.

98 John Harley Warner, ‘Therapeutic Explanation and the Edinburgh Bloodletting Controversy: Two Perspectives on the Medical Meaning of Science in the Mid-Nineteenth Century’, *Medical History* 24, 1980, 3, 241–58.

99 W. Rothstein, *American Physicians in the Nineteenth Century: From Sects to Science* (Baltimore, MD and London: Johns Hopkins University Press, 1972).

100 Warner, *Therapeutic Perspective*.

101 M.S.R Jenner et al. (eds), *Medicine and the Market in England and its Colonies, c. 1450–c. 1850* (London: Palgrave MacMillan, 2007), 1.

102 Codell Carter, *Decline of Therapeutic Bloodletting*, 7.

103 When mid-nineteenth-century doctors themselves explained why they rarely used bloodletting anymore, many relied on ‘change-of-type theory’. According to this explanation, people in the early nineteenth century often suffered from sthenic diseases, where the body became overly excited and overheated, and where bloodletting was a means of reducing harmful excess. However, toward the mid-1800s, such diseases had reportedly become rare. Doctors like Edinburgh professor William Alison claimed a dramatic increase in asthenic diseases, leaving patients in need of strengthening rather than depleting cures. The waning of therapeutic bleeding was therefore explained through which diseases were most common when a variant of this argument is to state that people’s bodily constitution had changed; whereas early nineteenth-century patients’ body types were easily inflamed, needing frequent bleeding, they later tended to be of a fragile, anaemic body type which could not stand to lose blood. By referring to changes in body type or disease prevalence, doctors could thus argue that bloodletting was a useful and legitimate practice without actually using it themselves, as it was only necessary to treat diseases not presently common. Warner, ‘Edinburgh Bloodletting Controversy’, 241–58. Warner references W. Alison, ‘Types of Fever and Blood-Letting’, *British Medical Journal*, 1865, 624; Charles Creighton, *A History of Epidemics in Britain*, 2 vols. (London: Cambridge University Press, 1894) 2:162–63; Charles Murchison, *A Treatise on the Continued Fevers of Great Britain* (London: Legare Street Press, 1873), 31.

104 See also Duden, *Woman Beneath the Skin*, 17.

105 Susan Sontag, *Illness as Metaphor* (New York, NY: Vintage Books, 1979), 61–62.

106 Martin, *The Woman in the Body*, 36.

107
*Ibid*., 34.

108
*Ibid*., 32.

109
*Ibid*., 35.

110
*Ibid*.

111 Maja Bondestam, ‘Om skägg, politik och behärskad manlighet’, in E. Heggestad ed, *En ny sits: Humaniora i förvandling: Vänbok till Margaretha Fahlgren* (Uppsala: Universitetsbiblioteket, 2008), 75.

112 Athena Vrettos, *Somatic Fictions: Imaging Illness in Victorian Culture* (Stanford, CA: Stanford University Press, 1995), 23.

113 Warner, ‘Therapeutic Explanation’, 253.

114 Clas Ekenstam, *Kroppens idéhistoria: Disciplinering och karaktärsdaning i Sverige 1700–1950* (Hedemora: Gidlunds, 1993), 135–49.

115
*Ibid*., 142.

116 Ben Baker-Benfield, ‘The Spermatic Economy: A Nineteenth Century View of Sexuality’, *Feminist Studies*, 1972, 1, 45–74.

117 Ekenstam, *Kroppens idéhistoria*, 143–44.

118
*Ibid*., 144. See also Martin, *Woman in the Body*, 34.

119 Ekenstam, *Kroppens idéhistoria*, 148.

120 Doctor Willén in Grästorp 1891.

121 Bondestam, ‘Om skägg’, 211.

122 Boel Berner, *Blodflöden: Blodgivning och blodtransfusion i det svenska samhället* (Halmstad: Arkiv Förlag, 2012), 19–56.

123
*Ibid*., 30.

124 E. Engdahl, ‘Esmarch: Blodbesparingsmetod vid operationer’ *Hygiea,* 1874, 36, 20–22.

125
*Ibid.*

126 Doctor Varenius in Hjo 1845.

